# Phosphopantetheinyl transferase binding and inhibition by amidino-urea and hydroxypyrimidinethione compounds

**DOI:** 10.1038/s41598-021-97197-4

**Published:** 2021-09-10

**Authors:** Coralie Carivenc, Laurent Maveyraud, Claire Blanger, Stéphanie Ballereau, Coralie Roy-Camille, Minh Chau Nguyen, Yves Génisson, Christophe Guilhot, Christian Chalut, Jean-Denis Pedelacq, Lionel Mourey

**Affiliations:** 1 Institut de Pharmacologie et de Biologie Structurale, IPBS, Université de Toulouse, CNRS, UPS, 31077 Toulouse, France; 2 Laboratoire de Synthèse et Physico-Chimie de Molécules d’Intérêt Biologique, LSPCMIB, Université de Toulouse, CNRS, UPS, 31062 Toulouse, France; 3 Present Address: Centre de Biochimie Structurale, CBS, CNRS, INSERM, Université de Montpellier, 34090 Montpellier, France; 4 Present Address: Evotec (France), 31100 Toulouse, France; 5 Present Address: Department of Pharmacology, University of Colorado School of Medicine, Aurora, CO USA

**Keywords:** Drug discovery, Biochemistry, Enzymes, Structural biology, Infectious diseases, Respiratory tract diseases

## Abstract

Owing to their role in activating enzymes essential for bacterial viability and pathogenicity, phosphopantetheinyl transferases represent novel and attractive drug targets. In this work, we examined the inhibitory effect of the aminido-urea 8918 compound against the phosphopantetheinyl transferases PptAb from *Mycobacterium abscessus* and PcpS from *Pseudomonas aeruginosa,* two pathogenic bacteria associated with cystic fibrosis and bronchiectasis, respectively. Compound 8918 exhibits inhibitory activity against PptAb but displays no activity against PcpS in vitro, while no antimicrobial activity against *Mycobacterium abscessus* or *Pseudomonas aeruginosa* could be detected. X-ray crystallographic analysis of 8918 bound to PptAb-CoA alone and in complex with an acyl carrier protein domain in addition to the crystal structure of PcpS in complex with CoA revealed the structural basis for the inhibition mechanism of PptAb by 8918 and its ineffectiveness against PcpS. Finally, *in crystallo* screening of potent inhibitors from the National Cancer Institute library identified a hydroxypyrimidinethione derivative that binds PptAb. Both compounds could serve as scaffolds for the future development of phosphopantetheinyl transferases inhibitors.

## Introduction

**Figure 1:**
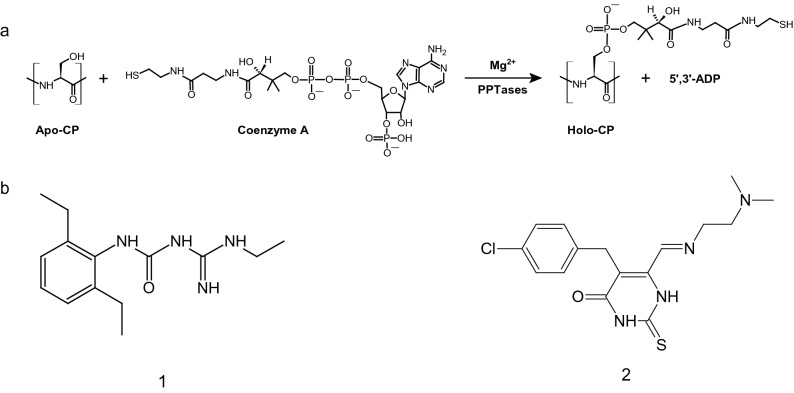
Phosphopantetheinylation plays a central role in metabolite biosynthetic pathways. (**a**) Reaction catalyzed by PPTases. (**b**) Inhibitors discussed in the present study. The aminido-urea 8918 inhibitor: (1-[(2,6-diethylphenyl)-3-N-ethylcarbamimodoyl]urea; (**1**)). The NCI compound P-62: (5-[(4-chlorophenyl)methyl]-6-[2-(dimethylamino)ethylimino-methyl]-2-sulfanylidene-1H-pyrimidin-4-one; (**2**)). This figure was generated using ACD/ChemSketch (Freeware) 2020.2.0 and Adobe Illustrator 25.4.1.

Phosphopantetheinyl transferases (PPTases) are essential enzymes involved in primary and secondary metabolite biosynthetic pathways^1^. They catalyze the transfer of the 4ʹ‐phosphopantetheinyl (Ppant) moiety of coenzyme A (CoA) to a strictly conserved serine residue of carrier protein (CP) domains present in fatty acid synthases (FASs), polyketide synthases (PKSs) and non-ribosomal peptide synthetases (NRPSs) (Fig. 1a). The covalent attachment of the 20 Å-long Ppant arm to CP domains allows tethering substrates and chemical intermediates through a reactive thioester bond and transferring them to the different catalytic centers of the synthases where enzymatic modifications can proceed. These synthases are involved in the biosynthesis of a broad range of natural lipids and complex organic compounds with excellent antifungal, antimicrobial, immunosuppressive, or antitumoral properties or which are essential for the virulence of major human bacterial pathogens.

*Mycobacterium tuberculosis*, the causative agent of tuberculosis, and related mycobacteria are unique among bacteria by possessing two types of FAS, FAS-I and FAS-II, and around 20 PKSs^2^. These megasynthases are involved in the biogenesis of the lipid-rich mycobacterial cell envelope that confers virulence, persistence, and drug resistance^3^. *M. tuberculosis* expresses two PPTases with non-redundant functions: MtAcpS, which activates FAS-I, and PptT, which activates all PKSs and NRPSs^4,5^ as well as AcpM^6^, which fuels substrates to the discrete enzymes making up FAS-II. It was previously shown that PptT expression is essential for the in vitro growth of the tubercle bacillus and for its viability in the mouse model during the acute and chronic phases of infection^4,7^. Altogether these findings indicate that PptT fulfils all the requirements to be a good drug target in the search for new anti-tuberculosis drugs^8^. Initiatives to inhibit PptT have emerged with the National Cancer Institute (NCI) Development Therapeutics Program (DTP) Open Compound collection (http://dtp.nci.nih.gov) using a combination of virtual screening, structure similarity-based searching, enzyme inhibition dose response with the MBP-PptT fusion protein^9^, and whole-cell inhibition of *M. tuberculosis* growth^10^. Thirty-six molecules were identified from primary and secondary screenings, with IC_50_ in the range 0.4–314 µM. As a general rule, none of these compounds displayed efficient whole-cell inhibition of *M. tuberculosis*. On the other hand, the discovery of the 8918 amidino-urea compound (1-[(2,6-diethylphenyl)-3-N-ethylcarbamimodoyl]urea; compound **1** in Fig. 1b) that kills *M. tuberculosis* by inhibiting PptT in vitro and in a mouse model, with no toxicity to human cells, contributed to redefine PPTases as major contributors to the development of antimycobacterial agents^11^. This motivated the recent development of a simple high-throughput colorimetric assay to directly screen for inhibitors of PptT^12^.

*Mycobacterium abscessus* and *Pseudomonas aeruginosa* represent two other human pathogens, involved in cystic fibrosis and bronchiectasis^13^, for which PPTases may represent attractive drug targets. *M. abscessus* produces two PPTases, which display high sequence identity with the PPTases from *M. tuberculosis*. Given the conservation of genes and biosynthetic pathways involved in the synthesis of lipids in mycobacteria, it is likely that the PptT ortholog from *M. abscessus*, PptAb, is required for the activation of diverse PKSs and NRPSs involved in the production of compounds such as mycolic acids, glycopeptidolipids and siderophores^14,15^. *P. aeruginosa* has a unique PPTase, PcpS, which was shown to be essential for the growth of the bacteria^16^. PcpS was found to activate the acyl carrier protein (ACP) component of the FAS system and also to act on NRPSs responsible for the assembly of pyoverdin and pyochelin, two siderophores required for the virulence of the bacteria^16,17^.

## Materials and methods

### Chemistry

(1-[(2,6-diethylphenyl)-3-N-ethylcarbamimodoyl]urea (8918; compound **1** in Fig. 1b) was prepared according to Ballinger *et al*^11^. To a solution of 1-ethylguanidine sulfate (300 mg, 1.62 mmol, 1.0 equiv) in acetone (6.5 mL) was added dropwise a 10% aqueous KOH solution (1.8 mL, 2.0 equiv). After stirring at RT for 5 min, 1,3-diethyl-2-isocyanatobenzene (304 mg, 1.8 mmol, 1.1 equiv) was added dropwise and the resulting solution allowed to reflux overnight. After cooling and removing of the solvents under a stream of nitrogen, deionized water (7 mL) was added to the crude reaction mixture and the pH adjusted to 7.0 using 2 N aqueous HCl. A white precipitate was collected by vacuum filtration and purified by chromatography on silica gel (ethyl acetate—petroleum ether 1:9 to 2:8) to give the title compound (154 mg, 37% yield) as a white solid. All analyses agreed with the data reported in the literature.

### Production and purification of recombinant proteins

PptAb and S2105A ACP were expressed and purified as previously described^18^. The *pcpS* gene was amplified by PCR from *P. aeruginosa* genomic DNA and cloned between the NdeI and HindIII restriction sites of the IPTG-inducible expression vector pET28a. The resulting plasmid expresses PcpS with an N-terminal His_6_ tag followed by a recognition site for thrombin. Frozen cells expressing the full-length PcpS were used to start an overnight 50 mL LB–kanamycin (35 µg mL^−1^) culture at 37 °C prior to inoculation in baffled flasks containing 2 L of the same media. Cells were allowed to grow for approximately 2 h at 37 °C before temperature was dropped from 37 to 30 °C. When OD_600_ reached 0.5–0.7, cells were induced with IPTG at a final concentration of 0.7 mM and grown for an additional 4 h prior to harvesting by centrifugation at 4,000 × *g* for 30 min. To eliminate residual LB medium, the cell pellets were resuspended in 40 mL of 100 mM HEPES pH 7.5, 150 mM NaCl prior to centrifugation at 4,000 × *g* for 20 min and storage at -80 °C. The pelleted cells were resuspended in 60 mL buffer A (50 mM HEPES pH 7.5, 150 mM NaCl, 1 mM MnCl_2_, 10% glycerol). After sonication (4 cycles of 30-s pulse, 50% amplitude, and power 6 W) and centrifugation at 30,000 × *g* for 30 min, the lysate was loaded on a gravity-flow column containing 2 mL of Talon Superflow Metal Affinity Resin (GE Healthcare). The His_6_-tagged PcpS was eluted from the column with buffer A supplemented with 120 mM imidazole. After elution, the His_6_ tag was removed by adding thrombin during 2 h dialysis against buffer C (25 mM HEPES pH 7.5, 50 mM NaCl, 5 mM MgCl_2_) at 4 °C. Dialysis product was then loaded again on metal affinity beads to remove free His_6_-tag and His_6_-tagged PcpS. The 10-mL protein flow-through was then concentrated to 5 mL and injected into a HiLoad Superdex 75 16/60 (GE Healthcare) preequilibrated with buffer C. After elution, the fractions of interest were supplemented with 2 mM CoA, pooled and concentrated to the desired concentration for crystallization.

### Activity/inhibition assay

Inhibition was assessed using the previously described activity assay^19^. Briefly, purified PPTases at 20 nM were incubated during 30 min at 4 °C with the desired concentration of compound, 8918 or NCI P-62, in 100% DMSO. Then 10 µM of ACP domain of PpsC and 50 μM of CoA were added to the reaction mixtures, also containing 5 mM MgCl_2_ and 1% DMSO, which were then incubated at 30 °C. Reactions were stopped at different time points by the addition of 100 mM EDTA. Samples were then loaded on a 10% polyacrylamide gel supplemented with 2.5 M urea and revealed with Coomassie blue. Blanks with or without DMSO were used as reference whereas apo-ACP alone was used as negative control.

### Inhibition of bacterial growth

The susceptibility of *M. abscessus*, *M. bovis* BCG and *P. aeruginosa* to 8918 was evaluated by using a colorimetric microassay based on the reduction of 3-(4,5-dimethylthiazol-2-yl)-2,5-diphenyltetrazolium bromide (MTT) (Sigma) to formazan by metabolically active cells^20–22^. A culture of *P. aeruginosa* grown in LB and cultures of *M. abscessus* and *M. bovis* BCG grown in 7H9/Tween80 were diluted in identical media to a DO_600_ of 0.05. 120 μL of bacterial suspension were transferred to 96-well plates in duplicate in the presence of 8918 at concentrations ranging from 0 et 128 μM. Plates were incubated at 37 °C for 4 h 20 (*P. aeruginosa*), 2 d (*M. abscessus*), or 4 d (*M. bovis* BCG). 30 μl of MTT solution (1 mg mL^−1^ in water) were added to individual wells and plates were further incubated at 37 °C for 30 min (*P. aeruginosa*), 6 h (*M. abscessus*), or 24 h (*M. bovis* BCG). 30 μL of lysis buffer (DMF/SDS 10% (1:2)) were added to each well and plates were incubated overnight at 37 °C. Absorbance values were measured at 570 nm using a plate reader (ClarioStar, BMG Labtech).

For time-kill kinetics assays, *M. abscessus* was grown in 25 mL of 7H9 medium with Tween 80 (0.05%) at 37 °C until OD_600_ reached 0.2. The culture was then divided into 5 subcultures of 5 mL before addition of 8918 (128, 64, or 32 μM) or P-62 (128 μM) diluted in DMSO, or DMSO alone. Cultures were incubated at 37 °C and the numbers of colony-forming units (CFU) were determined on days 0, 2, 4, and 7 by plating serial dilutions of each culture on 7H11 plates.

### Crystallization and structure determination of PptAb-CoA-8918 and PptAb-CoA-P-62

First, crystals of PptAb-CoA were grown at 12 °C using the sitting drop method by mixing an equivalent volume of protein solution at 10.0 mg mL^−1^ and of reservoir solution (0.1 MES pH 6.5, 20% PEG 8 K, 0.1 M Li_2_SO_4_). For obtaining the complex with 8918, PptAb-CoA crystals were soaked 20 h in a 5 mM solution of compound. Soaking with higher concentrations led to crystal cracking. For the crystallographic screening of compounds from the National Cancer Institute (NCI) Development Therapeutics Program (DTP) Open Compound collection, crystallization of PptAb-CoA was performed at 12 °C on a plate pre-coated with 1 μL of a 10 mM solution of compound in DMSO, as described in the ‘dry’ co-crystallization approach^23^. Crystals were directly cryo-cooled in liquid nitrogen. Data were collected at beamline ID30B at ESRF (compound 8918) and XALOC at ALBA (NCI compounds), and processed using XDS^24^ and CCP4^25^. Structures were initially refined using Pipedream^26^ followed by alternating refinement cycles with BUSTER^27^ and Phenix^28^ in order to allow for anisotropic B-factor refinement. Coot^29^ was used for model building and corrections. Refinement dictionaries for all compounds were generated using MarvinSketch (Marvin 20.8, 2020, ChemAxon) and the Grade server^30^.

### Crystallization and structure determination of PcpS-CoA

Crystals of PcpS-CoA were obtained at 12 °C using the hanging drop method by mixing an equivalent volume of freshly prepared protein solution at 6 mg mL^−1^ and of reservoir solution (0.1 M Na citrate pH 5.6, 10% PEG 4 K, 10% propanol). Crystals were directly cryo-cooled in liquid nitrogen. Data were collected at beamline XALOC at ALBA, and processed using XDS^24^ and Phenix^31^. Structures were solved by molecular replacement using the X-ray structure of PptT (PDB code 4U89) as a template in Phaser^32^. Iterative cycles of manual model building in Coot^29^ and refinement procedures using Phenix refine^28^ were applied until convergence.

### Crystallization and structure determination of PptAb-CoA-8918-ACP

First, 20 µL of PptAb-CoA at 405 µM were incubated with 0.8 µL of 8918 at 100 mM (PptAb-CoA:8918 ratio = 1:10) during 30 min at 4 °C. Then, 20 µL of the corresponding solution were mixed with 20 µL of S2105A ACP at 387 µM (PptAb-CoA-8918:S2105 ACP ratio = 1:1) and the mixture was further incubated for 30 min at 4 °C. Crystals were obtained using the hanging drop method at 12 °C by mixing an equivalent volume of the final complex solution and of reservoir solution (0.1 M Na Cacodylate pH 6.5, 30% PEG 8 K, 0.2 M (NH_4_)_2_SO_4_) and directly frozen in liquid nitrogen. Data were collected at beamline PX1 at SOLEIL, and processed using XDS^24^ and Phenix^31^. Structures were solved by molecular replacement using the X-ray structure of the PptAb-ACP complex (PDB code 6RCX) as a template in Phaser^32^. Refinement was conducted as described for PcpS-CoA.

## Results and discussion

### Inhibition of PPTase activity and bacterial growth by 8918

**Figure 2:**
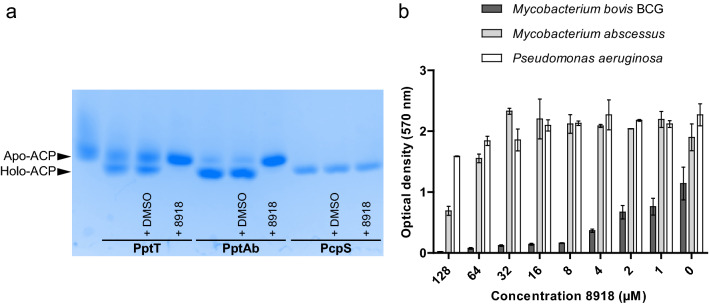
Inhibition by compound 8918. (**a**) In vitro conversion of apo- to holo-ACP by PptT, PptAb and PcpS after 60 min incubation with 5 µM 8918. The unlabeled lane corresponds to apo-ACP alone (used as a reference). (**b**) Bacterial viability assessed by MTT assay (serial dilution from 128 to 1 µM 8918). Results are mean and SD from duplicate samples. An uncropped version of the gel can be found in the Supplementary Information file (Supplementary Fig. 1). This figure was generated using Image Lab Version 6.1.0 (Bio-Rad Laboratories, Inc.) and Adobe Illustrator 25.4.1.

To check whether 8918 could inhibit the conversion of apo-ACP to holo-ACP by PptAb and PcpS, we resynthesized and evaluated this compound using an in vitro assay^18,19^. We found that 8918 inhibits ACP conversion by PptAb at nM concentrations and that total inhibition was achieved in the µM range (Fig. 2a and Supplementary Figs. S1 and S2). In contrast, no marked inhibition of PcpS could be observed even at high concentration of 8918 (5 µM; Fig. 2a and Supplementary Fig. S3), in accordance with previous work where 8918 was inactive against *P. aeruginosa*^11^. Here, we verified the lack of inhibitory activity of 8918 against *P. aeruginosa* by measuring the reduction of a tetrazolium salt by metabolically active cells into a colored formazan compound (Fig. 2b). We also assessed the impact of 8918 on the growth of two so far non-tested mycobacterial species, *M. bovis* BCG and *M. abscessus*. Surprisingly, although an inhibition by 8918 could be observed in the case of *M. bovis* BCG (Fig. 2b), in a manner reminiscent of what was observed in the case of *M. tuberculosis*^11^, no marked inhibition could be observed in the case of *M. abscessus* at concentration up to 128 μM (Fig. 2b). Consistently, a time-kill kinetics study showed that 8918 has no bactericidal activity against *M. abscessus* at concentrations up to 128 μM (Supplementary Fig. S4).

It has been shown that the inhibitory effect of 8918 against *M. tuberculosis* is potentiated by the PptH (phosphopantetheinyl hydrolase) enzyme encoded by the *rv2795c* gene^11^. PptH catalyzes the hydrolysis of the Ppant arm from holo-CP substrates and resistance to 8918 was in the majority associated with loss-of-function PptH mutations^11^. As other species in the Mycobacterium genus^33^, both *M. bovis* BCG and *M. abscessus* possess a PptH ortholog with identity score to the *M. tuberculosis* enzyme of 99 and 80%, respectively. We checked that the corresponding protein sequences also share the same active site residues, especially those proposed for the catalytic reaction, as PptH^33^. Then, the lack of inhibitory effect on the growth of *M. abscessus* does not seem to be linked to an inactive phosphopantetheinyl hydrolase ortholog. One hypothesis to explain this could be attributed to the low permeability of the *M. abscessus* cell envelope and consequently to a lower penetration of the inhibitor in bacteria.

### Crystallographic analysis of 8918 binding to PptAb

**Figure 3:**
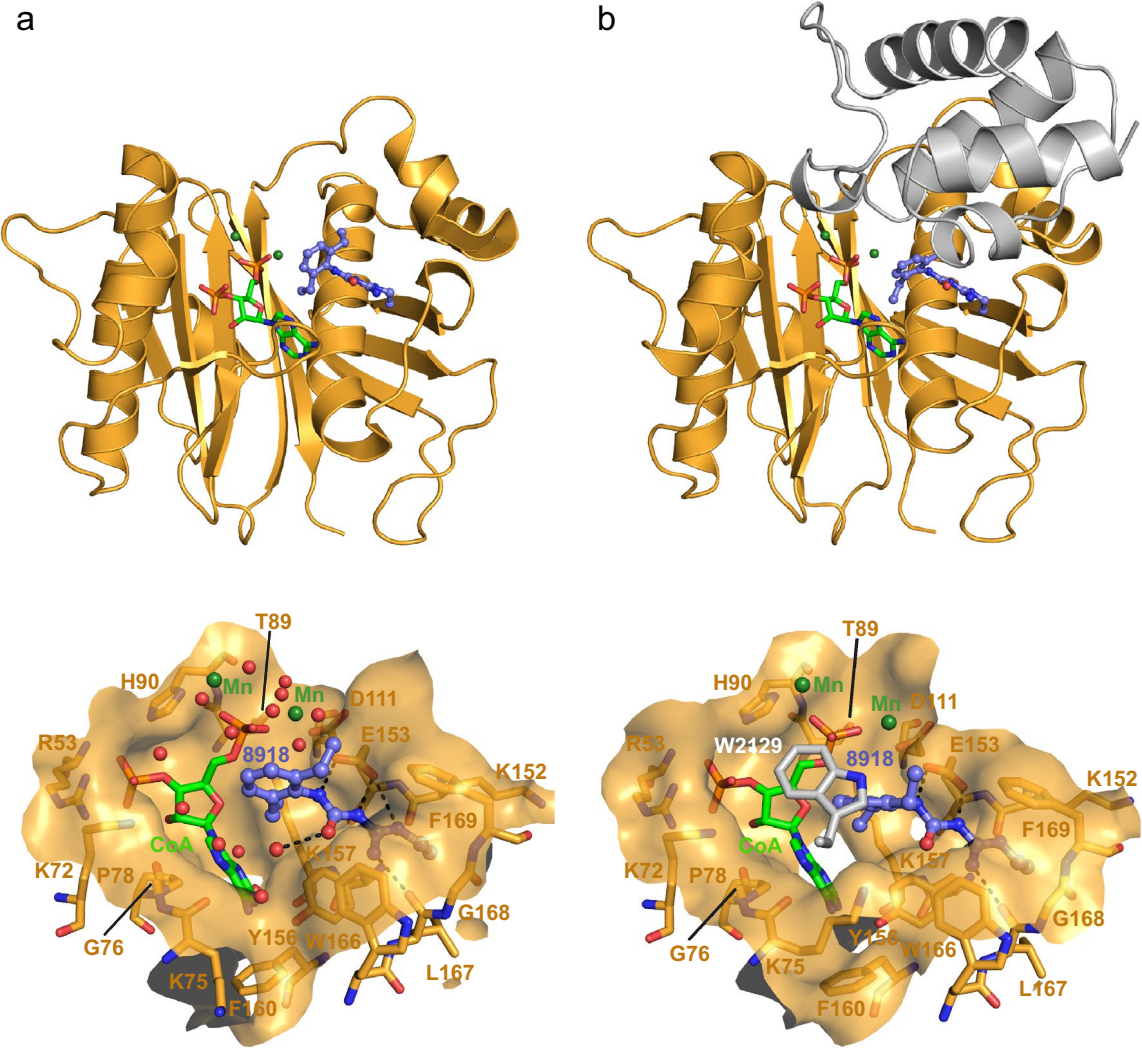
Crystallographic scrutinizing of CoA and 8918 binding to PptAb. X-rays structures of: (**a**) PptAb-CoA-8918 and (**b**) PptAb-CoA-8918-ACP. Top raw, overall view. Bottom raw, zoom-in the active site. Molecular surfaces include residues of PptAb within 5.0 Å of the ligands. Color scheme: PptAb, bright orange; ACP, dark grey; CoA and divalent cations, green; 8918, purple blue. Hydrogen bonds to 8918 are displayed as black dotted lines. This figure was generated using PyMol Version 2.4.1 (Schrödinger, LCC) and Adobe Illustrator 25.4.1. The 2D interaction maps were designed based on Ligplot+ v.2.2 (https://www.ebi.ac.uk/thornton-srv/software/LigPlus/).

**Figure 4:**
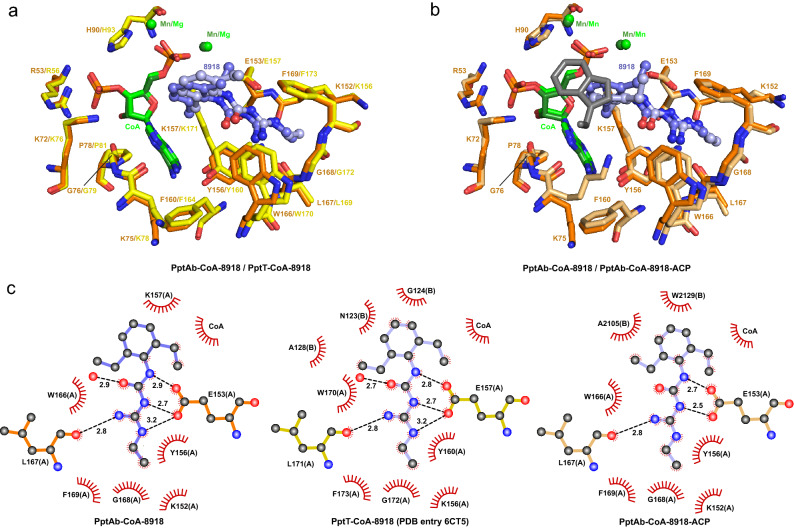
8918 binding to PptAb and PptT. (**a**) Superposition of PptAb-CoA-8918 (orange) and PptT-CoA-8918 (PDB entry 6CT5^11^, yellow). (**b**) Superposition of PptAb-CoA-8918 (orange) and PptAb-CoA-8918-ACP (light orange and grey). CoA and divalent cations are in green; 8918 is in purple blue. (**c**) 2D interaction maps: hydrogen bonds and their length are in black; protein residues/atoms and CoA involved in van der Waals contacts are represented by notched semicircles; letters in parentheses after residue names correspond to protein chain identifiers [one molecule, chain A, in the asymmetric unit of PptAb-CoA-8918; two molecules, chains A and B, for PptT-CoA-8918; for PptAb-CoA-8918-ACP, chains A and B are for PptAb and ACP, respectively]. This figure was generated using PyMol Version 2.4.1 (Schrödinger, LCC) and Adobe Illustrator 25.4.1. The 2D interaction maps were designed based on Ligplot+ v.2.2 (https://www.ebi.ac.uk/thornton-srv/software/LigPlus/).

Despite the absence of inhibition on *M. abscessus* growth, and since 8918 was able to inhibit ACP conversion in our in vitro assay, we wanted to confirm binding to PptAb using X-ray crystallography. Crystals of PptAb prepared in the presence of CoA and Mn^2+^ were soaked in a 5 mM solution of 8918 and the structure of the complex was solved at 1.40 Å (Supplementary Table S1 and Supplementary Fig. S5a; Fig. 3a). Interestingly, binding of compound 8918 to PptAb displays the same pattern as that observed for PptT (Fig. 4a,c). Three hydrogen bonds involving the catalytic base Glu153, one hydrogen bond formed with the main chain oxygen atom of Leu167, and van der Waals contacts established with Lys152, Tyr156, Lys157, Trp166, Gly168, Phe169, and CoA are found. It is noteworthy that a second 8918 molecule was found at the surface of PptT^11^, which is not the case for PptAb.

### 8918 Binding does not prevent formation of the PptAb-ACP complex

Based on our previous studies^18^, we have also been able to solve the 2.5 Å resolution X-ray structure of the complex formed between PptAb and its cognate ACP domain in the presence of CoA, Mn^2+^, and 8918 (Supplementary Table S1 and Supplementary Fig. S5b; Fig. 3b). Since PptAb was incubated with 4 mM 8918 prior to mixing with ACP, the inhibitor does not prevent the ACP domain and PptAb from interacting. In fact, the ACP domain intervenes very little in the interface. Only the residue Trp2129 is involved in binding through van der Waals contact and, by sitting on top of the diethylphenyl moiety of 8918, causing the displacement of the inhibitor along its longitudinal axis by about 0.9 Å (Fig. 4b). Despite this, three of the four hydrogen bonds observed for the enzyme alone are preserved (Figs. 3 and 4c).

### The crystal structure of PcpS explains the lack of inhibition by 8918

**Figure 5:**
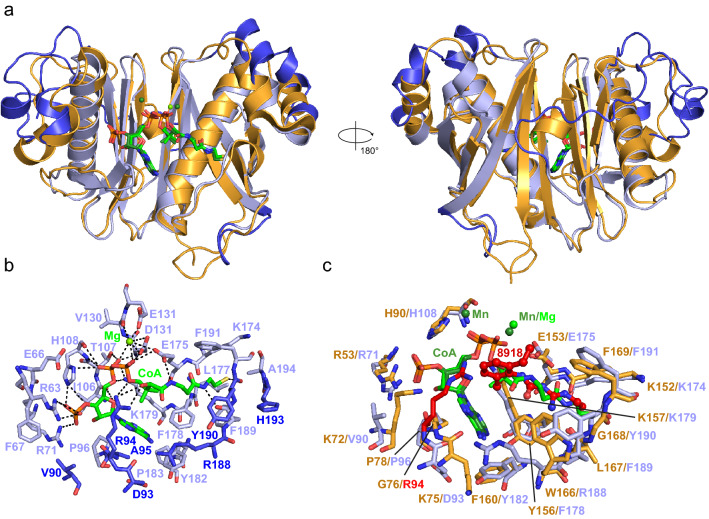
Structure of PcpS-CoA and its comparison with PptAb-CoA and PptAb-CoA-8918. (**a**) Overall superimposition of PcpS-CoA and PtpAb-CoA (PDB entry 6QW5)^18^. Two perpendicular views along the vertical axis are shown. (**b**) Zoom-in the active site of PcpS-CoA. Hydrogen bonds of CoA and Mg^2+^ with protein atoms and water molecules are in black (only direct interactions are displayed). (**c**) Superimposition of PptAb-CoA-8918 and PcpS-CoA. Color scheme: PptAb, bright orange; PcpS, lightblue; major differences between PcpS and PptAb (panels **a**,**c**), blue; CoA and divalent cations, green. In panel (**c**), R94 of PcpS-CoA and 8918 of PptAb-CoA-8918 are in red and numbering is for PptAb/PcpS. This figure was generated using PyMol Version 2.4.1 (Schrödinger, LCC) and Adobe Illustrator 25.4.1.

In the course of this study, we also resolved the crystal structure of PcpS in the presence of CoA at 2.40 Å resolution (Fig. 5a). PcpS crystallizes in space group P1 with four molecules in the asymmetric unit (Supplementary Table S1). However, analysis of the protein interfaces using PISA calculations as implemented on the EMBL-EBI server^34^ did not reveal any specific interactions that could result in the formation of stable high-order quaternary structures. Thus, the crystal structure confirms that PcpS is a monomeric Sfp-like PPTase (Sfp: surfactin phosphopantetheinyl transferase from *Bacillus subtilis*). This agrees with size exclusion chromatography and multi-angle light scattering (SEC-MALS) experiments performed on the purified protein, which gave an experimentally measured molar mass of 28,200 g mol^−1^ at the peak (to be compared to the calculated molar mass of 26.7 kDa). Structure-based sequence alignment indicates that PcpS have only 23 and 21% sequence identity with PptAb and PptT, respectively, whereas sequence identity between the latter amounts 65% (Supplementary Fig. S6). Despite low sequence identity, PcpS shares a high degree of structural conservation with the mycobacterial PPTases. For instance, superposing the structure of PcpS-CoA and that of PptAb-CoA (PDB entry 6QWU^18^) led to an RMSD value of 1.8 Å for 192 common Cα atoms. The main secondary structural elements that constitute the core of the PPTases are preserved whereas notable differences appear in the N-terminal part, which is longer in the case of PcpS, and in insertion and deletion elements, which are found in loops or short additional helices at the periphery of the protein (Fig. 5a and Supplementary Fig. S6). The cofactor in PcpS occupies the same position and configuration as those found for CoA in the PptAb structure with the Ppant arm perfectly visible in electron density (Supplementary Fig. S5c). The importance of pH, and in particular acidic pH, for the stabilization of the Ppant arm has been demonstrated^18^, and with that respect it should be noted that PcpS crystallizes in the presence of citrate buffer at pH 5.6. The chemical environments of cofactor and cations in PcpS and PptAb also share strong conservation with 55% sequence identity and 66% similarity among the 29 residues making up the ligand-binding site. Major differences are found at Lys72/Val90 (PptAb/PcpS numbering), Lys75/Asp93, Gly76/Arg94, Gln77/Ala95, Trp166/Arg188, Gly168/Tyr190, and Asp171/His193 (Fig. 5b,c). The Lys75 → Asp93, Gln77 → Ala95, Gly168 → Tyr190, and Asp171 → His193 PptAb to PcpS replacements do not seem to have any structural and chemical consequences, and it is noteworthy that the change Gly168 → Tyr190 compensates the loss of Trp at position 188. The Lys72 → Val90 substitution induces the loss of a hydrogen bond with CoA, which is more than compensated by the change of Gly76 in PptAb to Arg94 in PcpS (Fig. 5c). Tight binding of CoA to PcpS is completed by hydrogen bonds between Glu175, probably the PcpS catalytic base, to another oxygen of the β-phosphate and to the nitrogen atom of the β-alanine (Fig. 5b). Differences are also observed in the coordination of the M^2+^ ions. Two Mn^2+^ ions are found in the PptAb-CoA structure whereas only one Mg^2+^ has been identified looking at all four protein molecules constituting the PcpS-CoA asymmetric unit (Fig. 5c).

The presence of an arginine residue in position 94 and to a lesser extent in position 188 makes the PcpS active site less accessible, at least once the fixation of the cofactor is engaged. Despite several attempts, either by co-crystallization or by soaking, no structure of a complex between PcpS and 8918 could be obtained. In fact, the lack of in vitro inhibition and of *in crystallo* binding for PcpS can be explained by the structure. Indeed, Arg94 in PcpS, found at the same position as Gly76 in PptAb (Supplementary Fig. S6), a residue that takes part to the CoA-binding site, would cause a steric clash with the diethylphenyl group of the inhibitor (Fig. 5c). Although on the surface of the protein, the side chain of Arg94 offers very little flexibility, engaged as it is in hydrogen bonds with CoA (Fig. 5b). The fact that the Ppant of CoA is perfectly ordered in the structure of the complex with PcpS, which is not the case for PptAb-CoA-8918 ± ACP complexes (Supplementary Fig. S5) is in accordance with the finding that 8918 inhibits its target by displacing the Ppant arm, which decreases but does not abolish catalytic activity in the case of PptT^11^.

### Crystallographic screening of NCI compounds with PptT inhibitory effect

**Figure 6:**
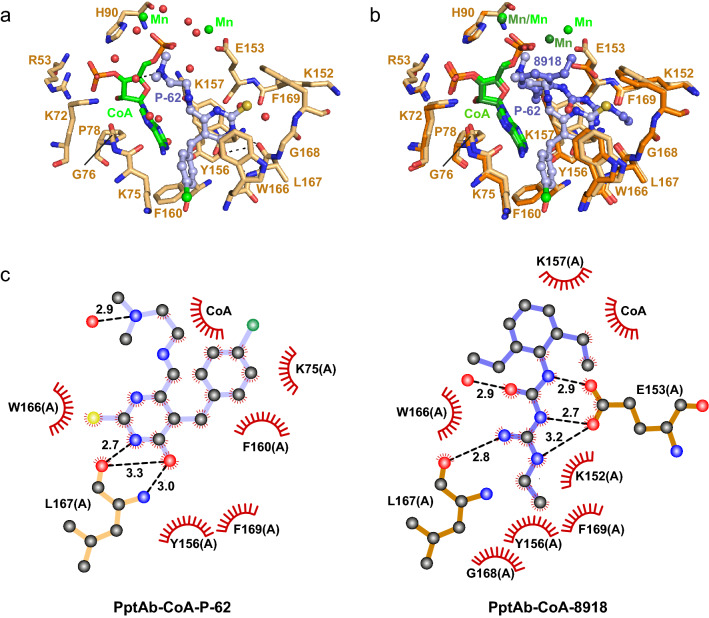
Crystallographic analysis of P-62 binding to PptAb-CoA. (**a**) Zoom-in the active site. (**b**) Superimposition of PptAb-CoA-P-62 (light orange) and PptAb-CoA-8918 (bright orange). CoA and divalent cations, green; P-62, light blue; 8918, purple blue. (**c**) 2D interaction map. Hydrogen bonds and their length are in black. Protein residues/atoms and CoA involved in van der Waals contacts are represented by notched semicircles. This figure was generated using PyMol Version 2.4.1 (Schrödinger, LCC) and Adobe Illustrator 25.4.1. The 2D interaction maps were designed based on Ligplot+ v.2.2 (https://www.ebi.ac.uk/thornton-srv/software/LigPlus/).

To check whether or not NCI compounds identified against PptT^10^ could also bind to PptAb, all 36 molecules were screened using the ‘dry’ co-crystallization approach^23^ and 24 of them gave crystals, 21 of which could be exploited for structure determination. Despite the high resolution of the collected data sets, only one compound, P-62 (5-[(4-chlorophenyl)methyl]-6-[2-(dimethylamino)ethylimino-methyl]-2-sulfanylidene-1H-pyrimidin-4-one; compound **2** in Fig. 1b) was found to bind to PptAb *in crystallo* (Supplementary Tables S1 and S2; Supplementary Fig. S5d). P-62 binding to PptAb displays significant similarity with that of 8918 (Fig. 6). The position and conformation of CoA and of all residues defining the ligand-binding site are conserved. One exception is for Glu153, whose side chain is flipped by 90° around the χ2 dihedral angle making polar interactions with one of the two Mn^2+^ ion. This ion is displaced by 2.0 Å when compared to its position in the PptAb-CoA-8918 complex. Residues Tyr156 (whose side chain is also flipped), Trp166 and Phe169 found to establish van der Waals contacts with 8918 also do so with P-62. The involvement of Leu167 main chain in hydrogen bonding to the ligand is also conserved. As for 8918, there is no electron density for the Ppant arm of CoA (Supplementary Fig. S5d), which is also disordered in the structure. Surprisingly no inhibition of the in vitro transfer by PptAb could be observed with P-62 (Supplementary Fig. S7) and P-62 neither affected cell viability of *M. abscessus* up to a 128 µM concentration (Supplementary Fig. S4).

In conclusion, molecules from the NCI database and an amidino-urea compound with proven inhibitory activity against PptT from *M. tuberculosis* were tested against *M. abscessus* PptAb in vitro and in broth culture. Although none of the NCI compounds exhibits an inhibitory activity against PptAb, inhibition of PptAb in the presence of compound 8918 could be achieved in the µM range. Nevertheless, whole cell based inhibition experiments of *M. abscessus* in broth culture also emphasize how *M. abscessus* represents an “antibiotic nightmare”^35,36^. Indeed, unlike *M. tuberculosis* for which the MIC_90_ of compound 8918 varies from 0.56 to 3 μM on clinical isolates, it has no impact on the growth of *M. abscessus* in broth culture at concentrations up to 128.0 μM, probably as a result of its inability to penetrate the cell envelope. Interestingly, binding of the NCI compound P-62 and 8918 to PptAb occupy the same region with interactions with conserved residues. Altogether, despite the limitations, this study shows that compounds of the amidinourea and hydroxypyrimidinethione type represent interesting molecular scaffolds for the rational design of more efficient inhibitors.

### Accession codes

The atomic coordinates and crystallographic structure factors of proteins and complexes described in this work have been deposited in the Protein Data Bank (www.rcsb.org) with accession codes as follows: PptAb-CoA-8918, 7B4R; PptAb-CoA-8918-ACP, 7BDW; PptAb-CoA-NCI-P62, 7B4S; PcpS-CoA, 7BCZ.

## Supplementary Information

Supplementary Information.

## References

[1] Beld J, Sonnenschein EC, Vickery CR, Noel JP, Burkart MD (2014). The phosphopantetheinyl transferases: Catalysis of a post-translational modification crucial for life. Nat. Prod. Rep..

[2] Kapopoulou A, Lew JM, Cole ST (2011). The MycoBrowser portal: A comprehensive and manually annotated resource for mycobacterial genomes. Tuberculosis (Edinb).

[3] Quadri LE (2014). Biosynthesis of mycobacterial lipids by polyketide synthases and beyond. Crit. Rev. Biochem. Mol. Biol..

[4] Chalut C, Botella L, de Sousa-D'Auria C, Houssin C, Guilhot C (2006). The nonredundant roles of two 4'-phosphopantetheinyl transferases in vital processes of Mycobacteria. Proc. Natl. Acad. Sci. U.S.A..

[5] Quadri LE, Sello J, Keating TA, Weinreb PH, Walsh CT (1998). Identification of a *Mycobacterium tuberculosis* gene cluster encoding the biosynthetic enzymes for assembly of the virulence-conferring siderophore mycobactin. Chem. Biol..

[6] Zimhony O (2015). AcpM, the meromycolate extension acyl carrier protein of *Mycobacterium tuberculosis*, is activated by the 4'-phosphopantetheinyl transferase PptT, a potential target of the multistep mycolic acid biosynthesis. Biochemistry.

[7] Leblanc C (2012). 4'-Phosphopantetheinyl transferase PptT, a new drug target required for *Mycobacterium tuberculosis* growth and persistence in vivo. PLoS Pathog..

[8] Huszár S, Chibale K, Singh V (2020). The quest for the holy grail: New antitubercular chemical entities, targets and strategies. Drug Discovery Today.

[9] Jung J (2014). Crystal structure of the essential *Mycobacterium tuberculosis* phosphopantetheinyl transferase PptT, solved as a fusion protein with maltose binding protein. J. Struct. Biol..

[10] Rohilla A, Khare G, Tyagi AK (2018). A combination of docking and cheminformatics approaches for the identification of inhibitors against 4′ phosphopantetheinyl transferase of *Mycobacterium tuberculosis*. RSC Adv..

[11] Ballinger E (2019). Opposing reactions in coenzyme A metabolism sensitize *Mycobacterium tuberculosis* to enzyme inhibition. Science.

[12] Brown AS, Owen JG, Jung J, Baker EN, Ackerley DF (2021). Inhibition of indigoidine synthesis as a high-throughput colourimetric screen for antibiotics targeting the essential *Mycobacterium tuberculosis* phosphopantetheinyl transferase PptT. Pharmaceutics.

[13] Johansen MD, Herrmann JL, Kremer L (2020). Non-tuberculous mycobacteria and the rise of *Mycobacterium abscessus*. Nat. Rev. Microbiol..

[14] Davidson LB, Nessar R, Kempaiah P, Perkins DJ, Byrd TF (2011). *Mycobacterium abscessus* glycopeptidolipid prevents respiratory epithelial TLR2 signaling as measured by HbetaD2 gene expression and IL-8 release. PLoS ONE.

[15] Nessar R, Reyrat JM, Davidson LB, Byrd TF (2011). Deletion of the mmpL4b gene in the *Mycobacterium abscessus* glycopeptidolipid biosynthetic pathway results in loss of surface colonization capability, but enhanced ability to replicate in human macrophages and stimulate their innate immune response. Microbiology.

[16] Barekzi N, Joshi S, Irwin S, Ontl T, Schweizer HP (2004). Genetic characterization of pcpS, encoding the multifunctional phosphopantetheinyl transferase of *Pseudomonas aeruginosa*. Microbiology.

[17] Finking R (2002). Characterization of a new type of phosphopantetheinyl transferase for fatty acid and siderophore synthesis in *Pseudomonas aeruginosa*. J. Biol. Chem..

[18] Nguyen MC (2020). Conformational flexibility of coenzyme A and its impact on the post-translational modification of acyl carrier proteins by 4'-phosphopantetheinyl transferases. FEBS J..

[19] Rottier K (2013). Detection of soluble co-factor dependent protein expression in vivo: application to the 4'-phosphopantetheinyl transferase PptT from *Mycobacterium tuberculosis*. J. Struct. Biol..

[20] Gomez-Flores R, Gupta S, Tamez-Guerra R, Mehta RT (1995). Determination of MICs for *Mycobacterium avium*-*M. intracellulare* complex in liquid medium by a colorimetric method. J. Clin. Microbiol..

[21] Foongladda S (2002). Rapid and simple MTT method for rifampicin and isoniazid susceptibility testing of *Mycobacterium tuberculosis*. Int. J. Tuberc. Lung Dis..

[22] Sankar MM, Gopinath K, Singla R, Singh S (2008). In-vitro antimycobacterial drug susceptibility testing of non-tubercular mycobacteria by tetrazolium microplate assay. Ann. Clin. Microbiol. Antimicrob..

[23] Gelin M (2015). Combining 'dry' co-crystallization and in situ diffraction to facilitate ligand screening by X-ray crystallography. Acta Crystallogr. D Biol. Crystallogr..

[24] Kabsch W (2010). XDS. Acta Crystallogr. D Biol. Crystallogr..

[25] Winn MD (2011). Overview of the CCP4 suite and current developments. Acta Crystallogr. D Biol. Crystallogr..

[26] Pipedream v. 1.2.5. (Global Phasing Ltd, 2011).

[27] BUSTER v. 2.10.3. (Global Phasing Ltd, 2017).

[28] Afonine PV (2012). Towards automated crystallographic structure refinement with phenix.refine. Acta Crystallogr. Sect..

[29] Emsley P, Lohkamp B, Scott WG, Cowtan K (2010). Features and development of Coot. Acta Crystallogr. D Biol. Crystallogr..

[30] Grade Web Server (Global Phasing Ltd, 2011).

[31] Liebschner D (2019). Macromolecular structure determination using X-rays, neutrons and electrons: Recent developments in Phenix. Acta Crystallogr. D Struct. Biol..

[32] McCoy AJ (2007). Phaser crystallographic software. J. Appl. Crystallogr..

[33] Mosior J, Bourland R, Soma S, Nathan C, Sacchettini J (2020). Structural insights into phosphopantetheinyl hydrolase PptH from *Mycobacterium tuberculosis*. Protein Sci..

[34] Krissinel E, Henrick K (2007). Inference of macromolecular assemblies from crystalline state. J. Mol. Biol..

[35] Lopeman RC, Harrison J, Desai M, Cox JAG (2019). *Mycobacterium abscessus*: Environmental bacterium turned clinical nightmare. Microorganisms.

[36] Nessar R, Cambau E, Reyrat JM, Murray A, Gicquel B (2012). *Mycobacterium abscessus*: A new antibiotic nightmare. J. Antimicrob. Chemother..

