# Supplementary material for: Phosphopantetheinyl transferase binding and inhibition by amidino-urea and hydroxypyrimidinethione compounds
Source: Sci Rep. 2021 Sep 10;11:18042. doi: 10.1038/s41598-021-97197-4 (PMC8433221; doi:10.1038/s41598-021-97197-4)
Supplement: Supplementary file 1 — Supplementary Information. [file 41598_2021_97197_MOESM1_ESM.pdf]

Supplementary information

Phosphopantetheinyl transferase binding and inhibition by amidino-urea and hydroxypyrimidinethione compounds

Coralie Carivenc[1,3,6], Laurent Maveyraud[1,6], Claire Blanger[2,4], Stéphanie Ballereau[2], Coralie Roy-Camille[1], Minh Chau Nguyen[1,5], Yves Génisson[2], Christophe Guilhot[1], Christian Chalut[1✉], Jean-Denis Pedelacq[1✉] and Lionel Mourey[1✉]

[1]Institut de Pharmacologie et de Biologie Structurale, IPBS, Université de Toulouse, CNRS, UPS, 31077 Toulouse, France. [2]Laboratoire de Synthèse et Physico-Chimie de Molécules d'Intérêt Biologique, LSPCMIB, Université de Toulouse, CNRS, UPS, 31062 Toulouse, France. [3]Present address: Centre de Biochimie Structurale, CBS, CNRS, INSERM, Université de Montpellier, 34090 Montpellier, France. [4]Present address: Evotec (France), 31100 Toulouse, France. [5]Present address: Department of Pharmacology, University of Colorado School of Medicine, Aurora, CO, USA. [6]These authors contributed equally: Coralie Carivenc and Laurent Maveyraud. ✉email: jean-denis.pedelacq@ipbs.fr; christian.chalut@ipbs.fr; lionel.mourey@ipbs.fr

Table of Contents

**S2-S3**	Supplementary figure legends
**S4**	Supplementary Table S1. Crystallographic data collection and refinement statistics.
**S5-S12**	Supplementary Table S2. *In crystallo* PptAb screening of NCI inhibitors against PptT activity.
**S13**	Supplementary references
**S14**	Supplementary Figure S1. Inhibition of PptT, PptAb and PcpS by compound 8918.
**S15**	Supplementary Figure S2. Conversion time-course of *apo*- to *holo*-ACP by *M. abscessus* PptAb after incubation with 8918.
**S16**	Supplementary Figure S3. Conversion time-course of *apo*- to *holo*-ACP by *P. aeruginosa* PcpS after incubation with 8918.
**S17**	Supplementary Figure S4. Time–kill curves of 8918 and P-62 against *M. abscessus*.
**S18**	Supplementary Figure S5. Crystallographic analysis of ligand binding to PptAb and PcpS.
**S19**	Supplementary Figure S6. Structure-based multiple sequence alignment of PptAb, PptT, and PcpS.
**S20**	Supplementary Figure S7. Conversion time-course of *apo*- to *holo*-ACP by *M. abscessus* PptAb after incubation with P-62.

Supplementary figure legends

Supplementary Figure S1. Inhibition of PptT, PptAb and PcpS by compound 8918. *In vitro* conversion of apo- to holo-ACP after 60 min incubation with 5 µM 8918. Uncropped gel corresponding to Figure 2a. This figure was generated using Image Lab Version 6.1.0 (Bio-Rad Laboratories, Inc.) and Adobe Illustrator 25.4.1.

Supplementary Figure S2. Conversion time-course of *apo*- to *holo*-ACP by *M. abscessus* PptAb after incubation with 8918. For each concentration, 4 time points corresponding to 0, 5, 15, 45 min were analyzed. Blanks with or without DMSO are displayed on the top left gel. Unlabeled lanes correspond to *apo*-ACP alone (used as a reference). Arrows indicate the positions of *apo*- and *holo*-ACP. All gels shown are uncropped. This figure was generated using Image Lab Version 6.1.0 (Bio-Rad Laboratories, Inc.) and Adobe Illustrator 25.4.1.

Supplementary Figure S3. Conversion time-course of *apo*- to *holo*-ACP by *P. aeruginosa* PcpS after incubation with 8918. For each concentration, 4 time points corresponding to 0, 5, 15, 45 min were analyzed. Blanks with or without DMSO are displayed on the top left gel. Unlabeled lanes correspond to *apo*-ACP alone (used as a reference). Arrows indicate the positions of *apo*- and *holo*-ACP. All gels shown are uncropped. This figure was generated using Image Lab Version 6.1.0 (Bio-Rad Laboratories, Inc.) and Adobe Illustrator 25.4.1.

Supplementary Figure S4. Time–kill curves of 8918 and P-62 against *M. abscessus*. Results are expressed as mean ± SD bacterial counts (log10 CFU mL^{-1}) measured in duplicate.

Supplementary Figure S5. Crystallographic analysis of ligand binding to PptAb and PcpS. (a) PptAb-CoA-8918. (b) PptAb-CoA-8918-ACP. (c) PcpS-CoA. (d) PptAb-CoA-P-62. Feature-enhanced maps (FEM) contoured at 1.0 σ are shown as blue mesh. The orientation is roughly the same as in Figure 3, bottom raw. Note that for sake of clarity, the side chain of ACP W2129 was not displayed in panel B. This figure was generated using PyMol Version 2.4.1 (Schrödinger, LCC) and Adobe Illustrator 25.4.1.

Supplementary Figure S6. Structure-based multiple sequence alignment of PptAb, PptT, and PcpS. The sequence numbering is for PptAb. Sequence homology is highlighted in red, whereas

sequence identity is shown as white letters on a red background. Aligned and unaligned residues are displayed in uppercase and lowercase, respectively, taking PptAb as reference. Residues absent from the structures are on a green background. Secondary structure elements as deduced from the corresponding crystal structures, i.e. PptAb-CoA-8918, PptT-CoA-8918 (PDB entry 6CT5), and PcpS-CoA are indicated. Residues of PptAb and PptT involved in 8918 binding are indicated by blue circles and stars for hydrophobic interactions and hydrogen bonds, respectively. Arg94 of PcpS is indicated by a red triangle. The sequence alignment was generated with ESPript 3[1].

Supplementary Figure S7. Conversion time-course of *apo*- to *holo*-ACP by *M. abscessus* PptAb after incubation with P-62. For each concentration, 4 time points corresponding to 0, 5, 15, 45 min were analyzed. Blanks with or without DMSO are displayed on the top left gel. Unlabeled lanes correspond to *apo*-ACP alone (used as a reference). Arrows indicate the positions of *apo*- and *holo*-ACP. All gels shown are uncropped. This figure was generated using Image Lab Version 6.1.0 (Bio-Rad Laboratories, Inc.) and Adobe Illustrator 25.4.1.

Supplementary Table S1. Crystallographic data collection and refinement statistics.

**Data set**	PptAb-CoA-8918	PptAb-CoA-8918-ACP	PptAb-CoA-P-62	PcpS-CoA
**PDB code**	7B4R	7BDW	7B4S	7BCZ
**Data collection**				
Beamline	ALBA-XALOC	SOLEIL-PX1	ESRF-ID30B	ALBA-XALOC
Space group	$P2_12_12$	$P2_12_12_1$	$P2_12_12$	$P1$
Unit cell *a*, *b*, *c* (Å) [α, β, γ (°)] for *P*1	62.96, 83.05, 56.02	50.84, 63.04, 108.57	62.84, 83.32, 56.09	44.36, 78.87, 81.12, 85.62, 84.62, 73.86
Resolution range (Å)[a]	46.44 – 1.40 (1.48 – 1.40)	39.57 – 2.55 (2.64 – 2.55)	46.53 – 1.38 (1.43 – 1.38)	42.49 – 2.40 (2.49 – 2.4)
No. of unique reflections	57,619 (9,112)	11,769 (1,055)	61,254 (5,937)	39,884 (3,949)
Completeness (%)	98.2 (97.5)	99.1 (91.7)	99.9 (99.9)	97.1 (96.5)
Redundancy	6.2 (6.3)	6.5 (6.5)	6.2 (6.1)	5.3 (5.3)
$<I/\sigma(I)>$	15.8 (1.3)	11.2 (1.6)	11.3 (0.6)	9.4 (2.1)
R_{sym} (%)	4.5 (108.7)	9.4 (82.7)	6.3 (260.3)	13.2 (71.0)
R_{meas} (%)	4.9 (118.0)	10.2 (89.7)	6.9 (285)	14.7 (78.9)
CC1/2	0.999 (0.835)	0.997 (0.784)	0.999 (0.486)	0.994 (0.784)
Wilson B factor (Å^2)	22.8	58.9	22.4	39.3
**Refinement**				
No. of reflections (work / test)	57,514 (2,908)	11,761 (1,055)	60,275 (3,058)	39,857 (3,949)
R_{work} / R_{free}	0.1618 / 0.1943	0.2120 / 0.2545	0.1685 / 0.1948	0.2113 / 0.2603
No. of molecules/AU[b]	1	1 PptAb and 1 ACP	1	4
No. of non-hydrogen atoms	2,083	2,290	2,166	7,843
Protein	1,769	2,234	1,790	7,488
Ligands	46	46	50	192
Ions	2 Mn^{2+}	2 Mn^{2+}	2 Mn^{2+}	1 Mg^{2+}
Water molecules	266	8	324	161
RMS deviations				
Bond lengths (Å)	0.014	0.002	0.012	0.013
Bond angles (°)	1.384	0.520	1.397	0.960
Ramachandran plot (%)				
Most favored	99.5	96.9	99.1	96.3
Allowed / disallowed	0.5 / 0	3.1 / 0	0.9 / 0	3.7 / 0
Average B-factors (Å^2)	32.2	81.7	31.5	48.0
Protein	30.6	81.9	29.5	48.2
Ligands	30.4	73.8	31.7	42.2
Solvent	43.3	62.3	42.8	44.2

[a]Values in brackets are for the highest-resolution shell.
[b]Asymmetric unit

Supplementary Table S2. ***In crystallo*** **PptAb screening of NCI inhibitors against PptT activity.**

**Compound**[a]	**2D structure / Name**	**IC$_{50}$**[b] **(μg mL^{-1} / μM)**	**Crystal**	**Collect**	**Resolution (Å)**	**Ligand binding**
P-4/23925<br>-	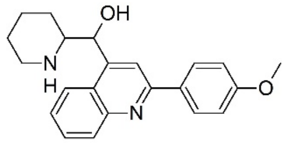<br>[2-(4-methoxyphenyl)quinolin-4-yl](piperidin-2-yl)methanol	68.2 / 162.0	Yes	Yes	1.75	No
P-52/53979<br>7155-11-5	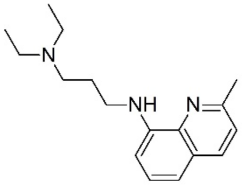<br>N',N'-diethyl-N-(2-methylquinolin-8-yl)propane-1,3-diamine	2.3 / 4.4	Yes	No	-	-
P-61/153785<br>31349-12-9	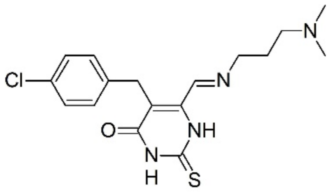<br>5-[(4-chlorophenyl)methyl]-6-[3-(dimethylamino)propyliminomethyl]-2-sulfanylidene-1H-pyrimidin-4-one	10.1 / 27.8	Yes	Yes	1.53	No
P-62/153786<br>31349-11-8	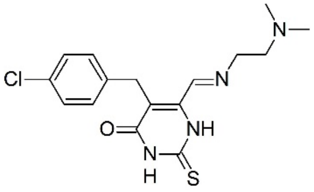<br>5-[(4-chlorophenyl)methyl]-6-[2-(dimethylamino)ethyliminomethyl]-2-sulfanylidene-1H-pyrimidin-4-one	5.8 / 16.6	Yes	Yes	1.38	Yes

Supplementary Table S2. ***In crystallo*** **PptAb screening of NCI inhibitors against PptT activity (continued).**

**Compound[a]**	**2D structure / Name**	**IC_{50}[b] (μg mL^{-1} / μM)**	**Crystal**	**Collect**	**Resolution (Å)**	**Ligand binding**
P-70/305817<br>57120-44-2	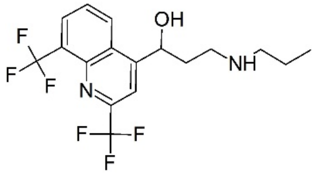<br>1-[2,8-bis(trifluoromethyl)quinolin-4-yl]-3-(propylamino)propan-1-ol	7.9 / 18.9	No	No	-	-
P-81/659178<br>-	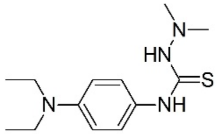<br>N-[4-(diethylamino)phenyl]-2,2-dimethylhydrazine-1-carbothioamide	6.6 / 24.8	No	No	-	-
P-86/8544<br>-	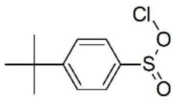<br>1-tert-butyl-4-[(chlorooxy)sulfinyl]benzene	2.7 / 11.4	No	No	-	-
P-94/47908<br>6638-10-4	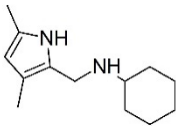<br>N-[(3,5-dimethyl-1H-pyrrol-2-yl)methyl]cyclohexanamine	22.0 / 106.8	Yes	Yes	1.52	No
P-95/48602<br>-	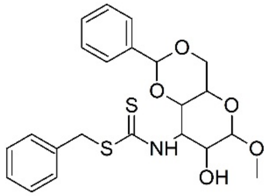<br>Methyl 4, 6-O-benzylidene-3-{[(benzylsulfanyl) carbonothioyl]amino}-3-deoxyhexopyranoside	8.7 / 19.5	Yes	Yes	1.42	No

Supplementary Table S2. ***In crystallo*** **PptAb screening of NCI inhibitors against PptT activity (continued).**

**Compound**[a]	**2D structure / Name**	**IC_{50}**[b] **(µg mL^{-1} / µM)**	**Crystal**	**Collect**	**Resolution (Å)**	**Ligand binding**
P-102/95701<br>16434-97-2	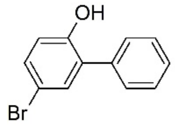<br>5-bromo[1,1'-biphenyl]-2-ol	43.1 / 173.0	Not tested	-	-	-
P-103/112665	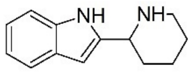<br>2-piperidin-2-yl-indole	26.0 / 130.0	Yes	Yes	1.90	No
P-110/138367<br>23817-85-8	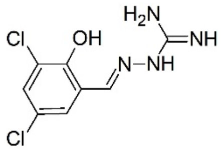<br>2-[(3,5-dichloro-2-hydroxyphenyl)methylidene]hydrazine-1-carboximidamide	8.5 / 34.6	Yes	Yes	1.51	No
P-114/201529<br>-	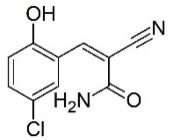<br>3-(5-chloro-2-hydroxyphenyl)-2-cyanoprop-2-enamide	18.9 / 84.5	Yes	Yes	1.67	No
P-116/208696<br>16901-38-5	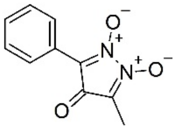<br>3-methyl-1,2-dioxido-5-phenylpyrazole-1,2-diium-4-one	12.8 / 62.7	Yes	Yes	1.58	No

Supplementary Table S2. ***In crystallo*** **PptAb screening of NCI inhibitors against PptT activity (continued).**

**Compound[a]**	**2D structure / Name**	**IC$_{50}$[b] (μg mL^{-1} / μM)**	**Crystal**	**Collect**	**Resolution (Å)**	**Ligand binding**
P-137/80978<br>-	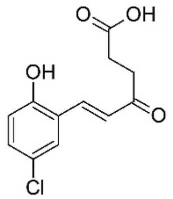<br>6-(5-chloro-2-hydroxyphenyl)-4-oxohex-5-enoic acid	1.2 / 4.8	No	No	-	-
P-138/81164<br>537-09-7	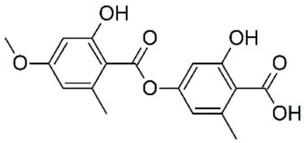<br>2-hydroxy-4-[(2-hydroxy-4-methoxy-6-methylbenzoyl)oxy]-6-methylbenzoic acid (evernic acid)	18.3 / 55.1	No	No	-	-
P-147/114808<br>-	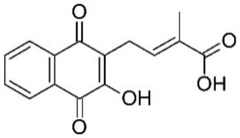<br>4-(3-hydroxy-1,4-dioxo-1,4-dihydronaphthalen-2-yl)-2-methylbut-2-enoic acid	1.4 / 5.2	Yes	Yes	1.75	No
P177/18762<br>5453-55-4	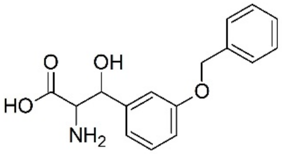<br>2-amino-3-[3-(benzyloxy)phenyl]-3-hydroxypropanoic acid	3.6 / 12.5	No	No	-	-
P-183/52238<br>7147-65-1	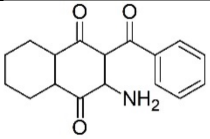<br>2-amino-3-benzoyloctahydronaphthalene-1,4-dione	1.2 / 4.1	Yes	Yes	1.31	No

Supplementary Table S2. ***In crystallo*** **PptAb screening of NCI inhibitors against PptT activity (continued).**

Compound[a]	2D structure / Name	IC_{50}[b] (μg mL^{-1} / μM)	Crystal	Collect	Resolution (Å)	Ligand binding
P-215/665278<br>-	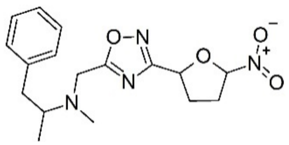<br>N-methyl-N-{[3-(5-nitrooxolan-2-yl)-1,2,4-oxadiazol-5-yl]methyl}-1-phenylpropan-2-amine	7.7 / 22.2	Yes	Yes	1.42	No
PS-9/16001<br>5437-31-0	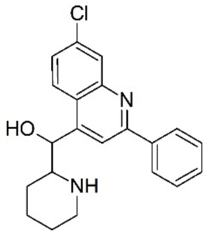<br>(7-chloro-2-phenylquinolin-4-yl)(piperidin-2-yl)methanol	64.5 / 151.4	No	No	-	-
PS-4/13040<br>5427-49-6	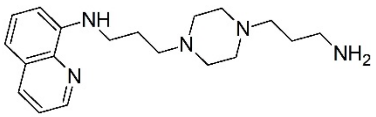<br>N-[3-[4-(3-aminopropyl)piperazin-1-yl]propyl]quinolin-8-amine	0.3 / 0.6	Yes	No	-	-
PS-8/14893<br>-	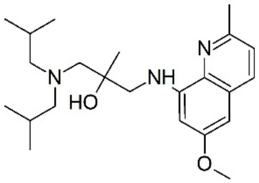<br>1-(Diisobutylamino)-3-[(6-methoxy-2-methyl-8-quinolinyl)amino]-2-methyl-2-propanol	0.6 / 1.4	No	No	-	-

Supplementary Table S2. ***In crystallo*** **PptAb screening of NCI inhibitors against PptT activity (continued).**

**Compound**[a]	**2D structure / Name**	**IC_{50}**[b]<br>**(μg mL^{-1} / μM)**	**Crystal**	**Collect**	**Resolution (Å)**	**Ligand binding**
PS-10/29962<br>6324-37-4	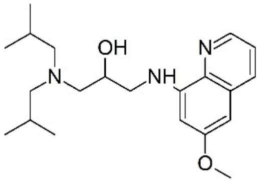<br>1-(Diisobutylamino)-3-[(6-methoxy-2-methyl-8-quinolinyl)amino]-2-propanol	0.6 / 1.3	No	No	-	-
PS-14/53975<br>-	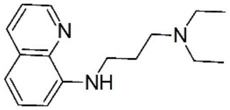<br>N,N-diethyl-N'-(quinolin-8-yl)propane-1,3-diamine	0.7 / 2.2	No	No	-	-
PS-15/53976<br>7253-89-6	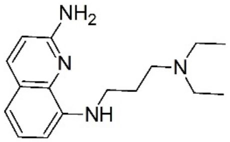<br>N8-[3-(diethylamino)propyl]quinoline-2,8-diamine	2.9 / 5.4	Yes	Yes	1.40	No
PS-40/328398<br>-	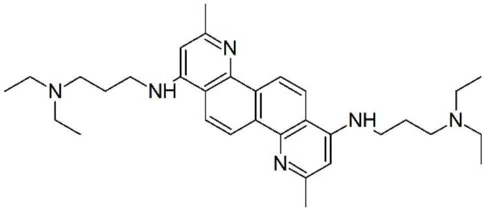<br>N,N'-Bis[3-(diethylamino)propyl]-3,9-dimethylquinolino[8,7-h] quinoline-1,7-diamine	0.2 / 0.4	No	No	-	-

Supplementary Table S2. ***In crystallo*** **PptAb screening of NCI inhibitors against PptT activity (continued).**

**Compound**[a]	**2D structure / Name**	**IC$_{50}$**[b] **(μg mL^{-1} / μM)**	**Crystal**	**Collect**	**Resolution (Å)**	**Ligand binding**
PS-37/305815<br>57120-41-9	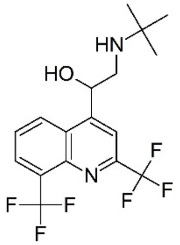<br>1-[2,8-bis(trifluoromethyl)quinolin-4-yl]-2-(tert-butylamino)ethanol	29.9 / 71.8	Yes	Yes	1.52	No
PS-39/305830<br>-	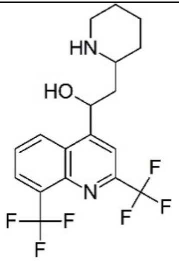<br>1-[2,8-bis(trifluoromethyl)quinolin-4-yl]-2-(piperidin-2-yl)ethan-1-ol	34.5 / 80.4	Yes	Yes	2.06	No
PS-3/9772<br>13185-00-7	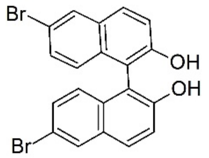<br>6,6'-dibromo[1,1'-binaphthalene]-2,2'-diol	4.1 / 9.3	Yes	Yes	2.13	No
PS-13/53508<br>4400-05-9	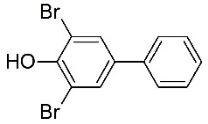<br>3,5-dibromo[1,1'-biphenyl]-4-ol	11.1 / 34.0	Yes	Yes	1.34	No

Supplementary Table S2. ***In crystallo*** **PptAb screening of NCI inhibitors against PptT activity (continued and end).**

**Compound[a]**	**2D structure / Name**	**IC_{50}[b] (µg mL^{-1} / µM)**	**Crystal**	**Collect**	**Resolution (Å)**	**Ligand binding**
PS-24/95696<br>4544-71-2	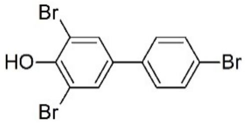<br>3,4',5-tribromo[1,1'-biphenyl]-4-ol	5.0 / 12.3	Yes	Yes	1.22	No
PS-25/95809<br>55815-20-8	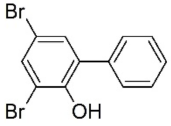<br>3,5-dibromo[1,1'-biphenyl]-2-ol	18.0 / 54.9	Yes	Yes	1.40	No
PS-31/130468<br>29558-77-8	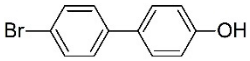<br>4'-bromo[1,1'-biphenyl]-4-ol	7.1 / 28.6	Yes	Yes	1.96	No
PS-18/65385<br>15996-57-3	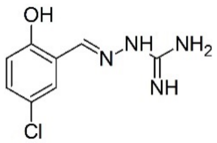<br>2-[(5-chloro-2-hydroxyphenyl)methylidene]hydrazine-1-carboximidamide	66.8 / 313.6	Yes	Yes	1.66	No
PS-19/67610<br>13308-87-7	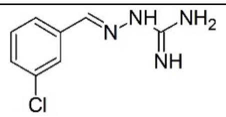<br>2-[(3-chlorophenyl)methylidene]hydrazine-1-carboximidamide	37.6 / 190.9	Yes	No	-	-

[a]First line: numbering from [2]/NSC number from the National Cancer Institute (NCI) Development Therapeutics Program Open Compound collection; second line: CAS number when available.
[b]Inhibition against PptT. Values in µg/ml were taken from [2] and converted in µM.

Supplementary references

1. Robert, X. & Gouet, P. Deciphering key features in protein structures with the new ENDscript server. *Nucleic Acids Res* **42**(Web Server issue), W320-324 (2014).
2. Rohilla, A., Khare, G. & Tyagi, A. K. A combination of docking and cheminformatics approaches for the identification of inhibitors against 4′ phosphopantetheinyl transferase of Mycobacterium tuberculosis. *RSC Advances* **8**(1), 328-341 (2018).

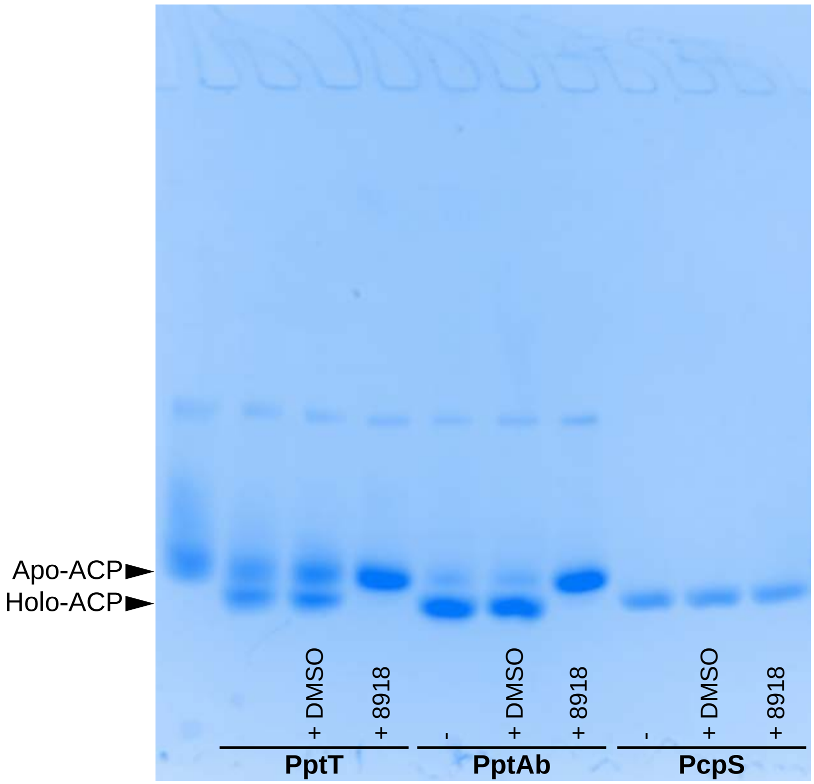

Supplementary Figure S1

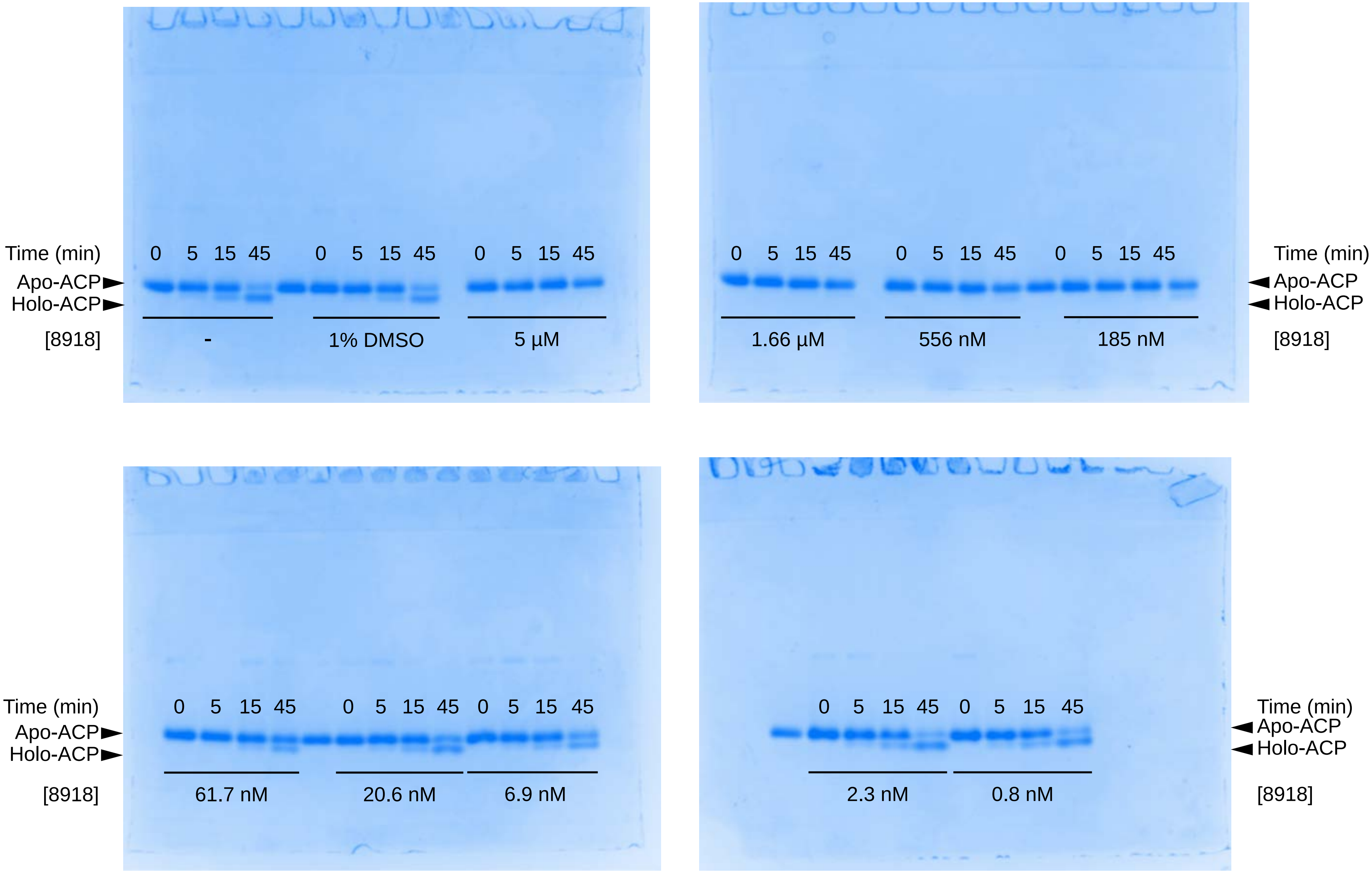

Supplementary Figure S2

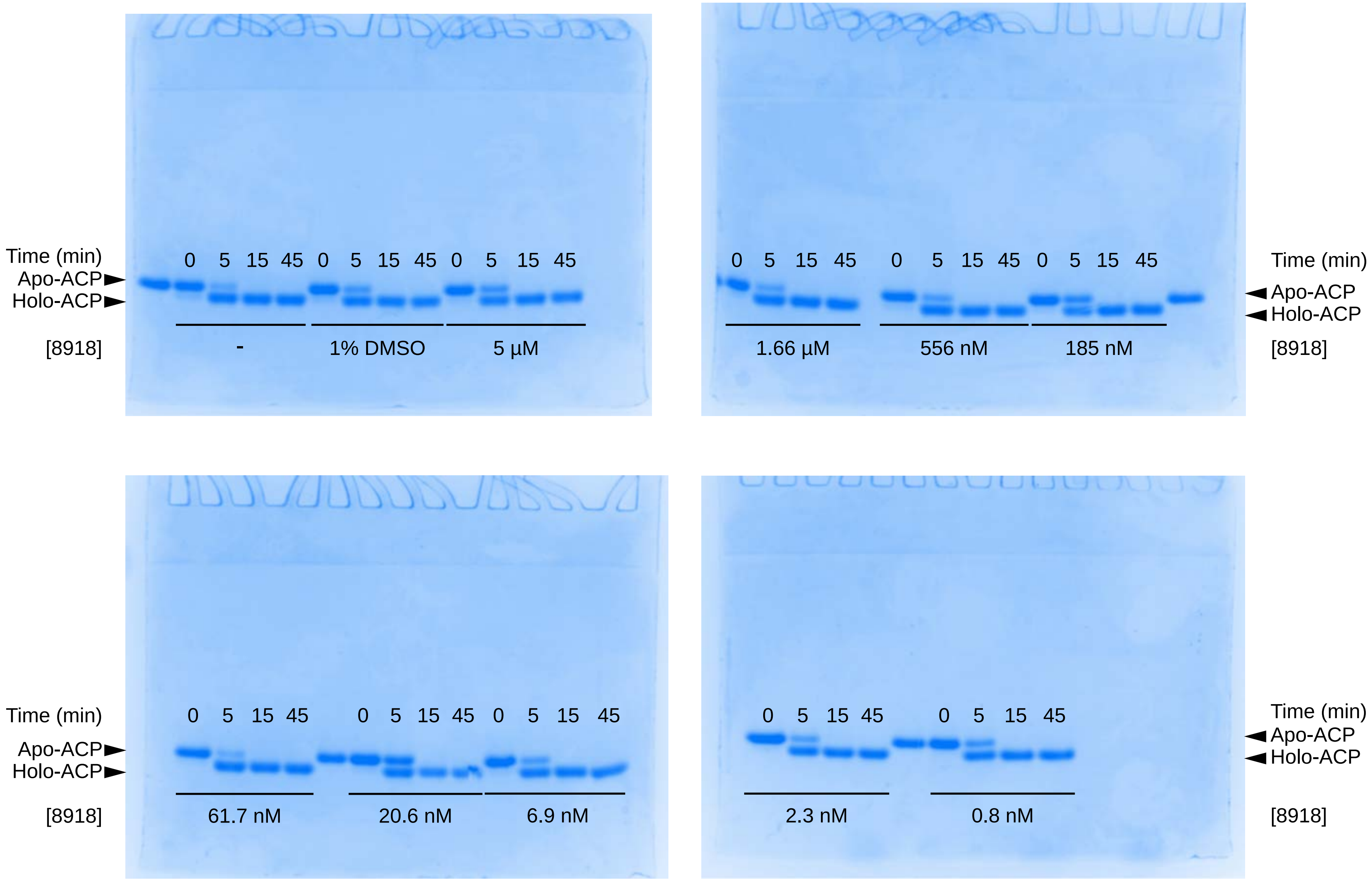

Supplementary Figure S3

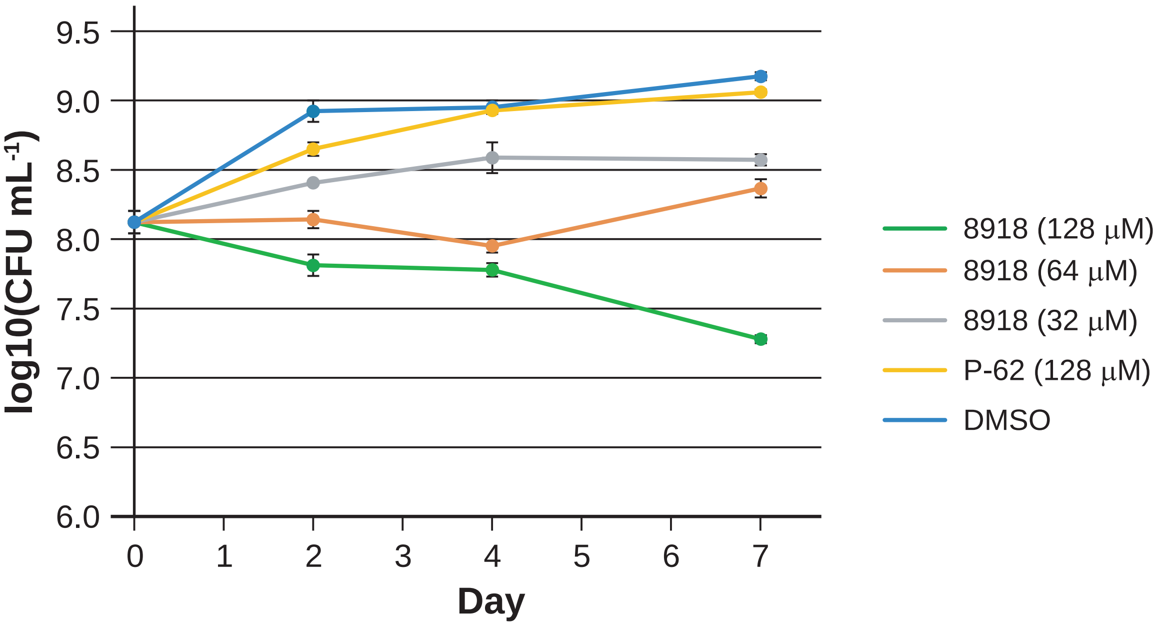

Supplementary Figure S4

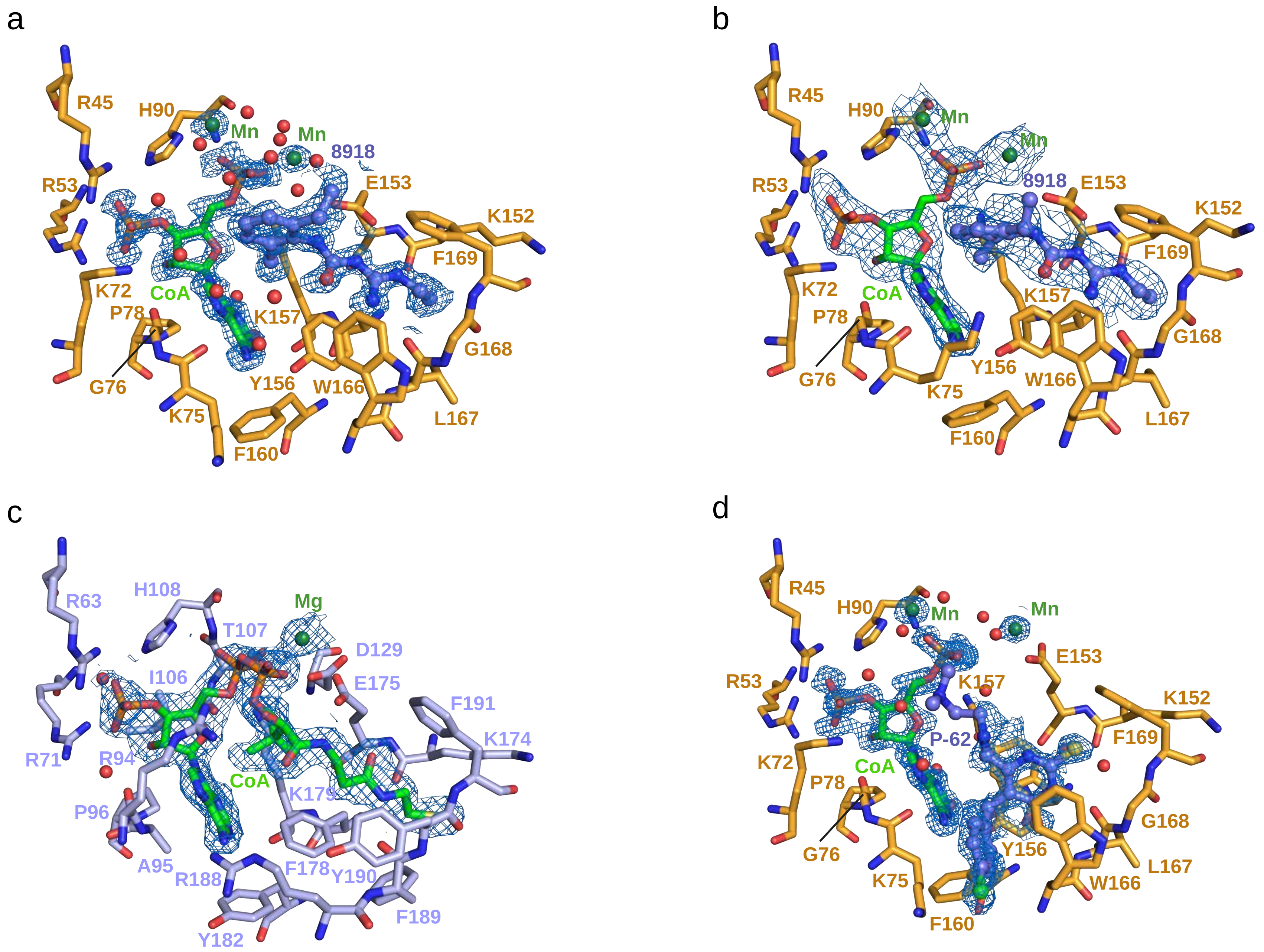

Supplementary Figure S5

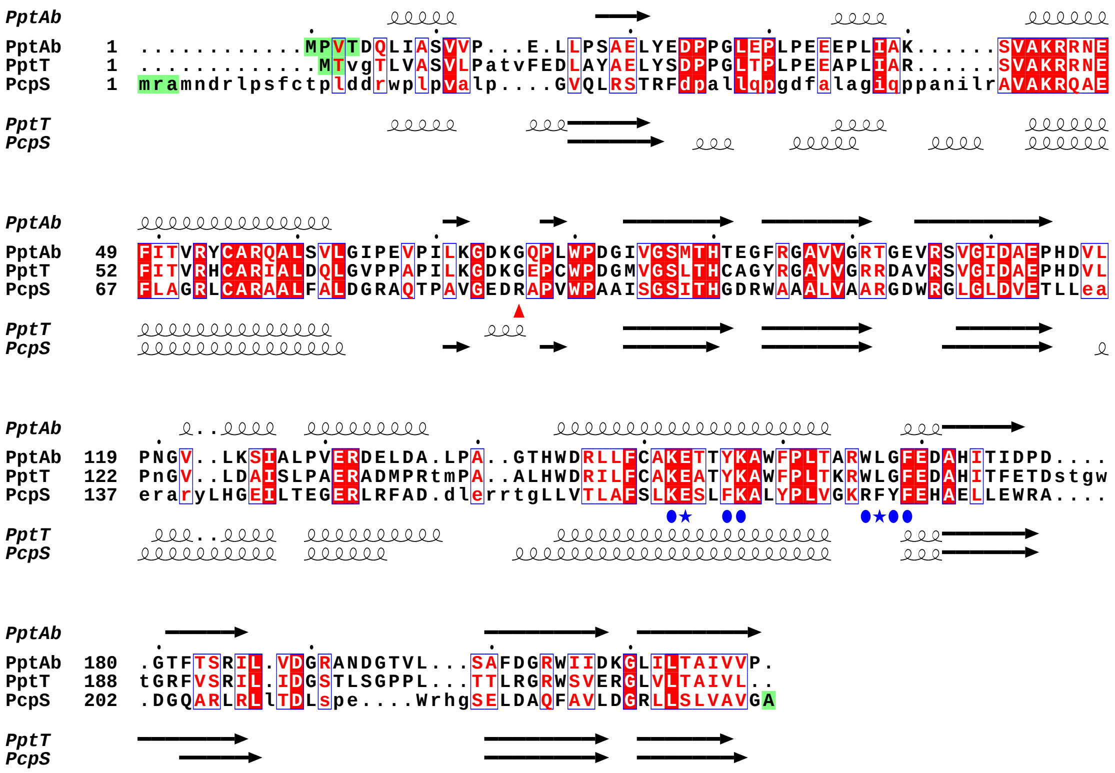

Supplementary Figure S6

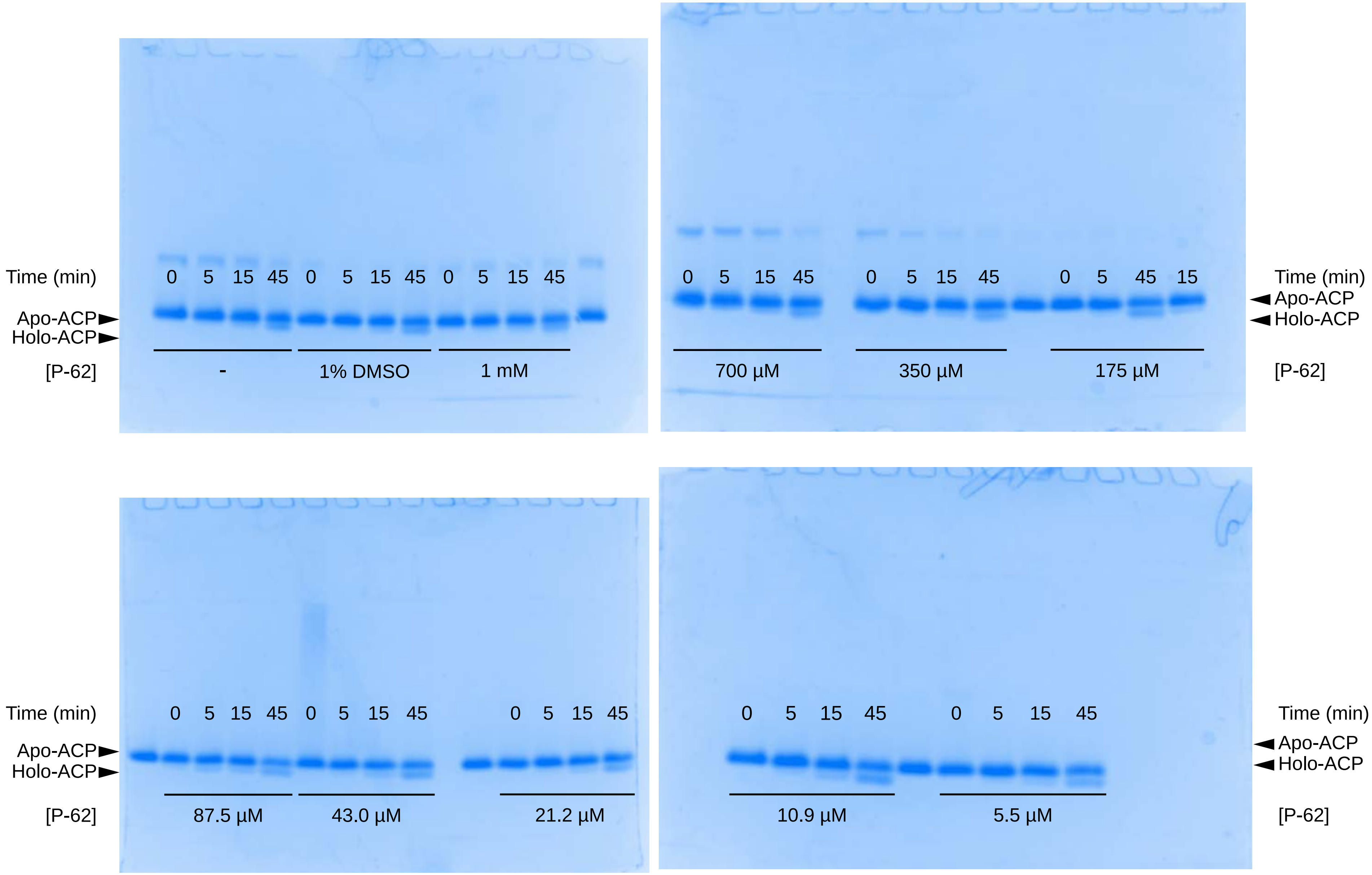

Supplementary Figure S7
